# Microwave assisted synthesis of ZnO-PbS heterojuction for degradation of organic pollutants under visible light

**DOI:** 10.1038/s41598-020-59066-4

**Published:** 2020-02-10

**Authors:** Ganapathy Mano, Subramanian Harinee, Sampath Sridhar, Mahalingam Ashok, Alagan Viswanathan

**Affiliations:** 1 Department of Physics, University College of Engineering Bharathidasan Institute of Technology (BIT-Campus), Anna University, Tiruchirappalli, 620024 Tamil Nadu India; 2 Department of Environmental Engineering, National Ilan University, Yilan, Taiwan; 3 Department of Physics, National Institute of Technology, Tiruchirappalli, 620015 Tamil Nadu India; 4 Department of Physics, Vel Tech Rangarajan Dr. Sagunthala R&D Institute of Science and Technology, Avadi, Chennai, 600062 Tamil Nadu India

**Keywords:** Photocatalysis, Synthesis and processing

## Abstract

ZnO, PbS and ZnO-PbS heterojunction were prepared by microwave irradiation to improve the organic pollutants degradation under visible light irradiation. Hexagonal (wurtzite) and cubic crystal structure of ZnO and PbS respectively were confirmed by PXRD. Nano-plate, nano-sponge and nano-sponge imprinted over nano-sheet like morphology of ZnO, PbS and ZnO-PbS respectively were revealed through FESEM analysis. HR-TEM analysis provides the formation of heterojunction. XPS analysis shows the presence of the ZnO-PbS heterojunction. UV-Visible spectroscopy confirms the enhanced visible light response of ZnO-PbS heterojunction than the bare ZnO. The PL and EIS results indicate ZnO-PbS heterojunction exhibited lowest recombination of excitons and electron transfer resistance. Synergistic effect of ZnO-PbS heterojunction leads to efficient degradation against organic pollutants than bare ZnO and PbS. Aniline and formaldehyde were successfully degraded around 95% and 79% respectively, under solar light irradiation. As-prepared photocatalysts obeys pseudo first order reaction kinetics. HPLC analysis also confirms the successful mineralization of organic pollutants into water and CO_2_.

## Introduction

Increasing the population growth demands various energy production and consumption which derives different form of toxic effluents. Therefore, the removal of the toxic effluents is important to control the pollution. In recent years, semiconductor mediated heterogeneous photocatalysis becoming promising technique to eliminate or covert toxic into non-toxic compounds. Further, they consist additional potential properties such as non-toxicity, photochemical stability, energy conversion and low cost^1–5^. In recent years different kinds of semiconductors are employed for photocatalytic application such as ZnO, TiO_2_, CdS, ZnS, WO_3_, SnO_2_, SrTiO_3_, YFeO_3_ etc. Among the semiconductor based photocatalyst, ZnO and TiO_2_ are widely used due to their strong oxidation capacity, chemical stability, non-toxicity, low cost etc. ZnO (3.37 eV) is one of the best alternate semiconductor material for TiO_2_ (3.2 eV) as it has similar band gap energy and high quantum efficiency^6–18^.

ZnO photocatalyst is highly active under UV region in the solar spectrum, fast recombination of photo generated electron-hole pairs and photo corrosion properties^19^. In general, the photocatalytic efficiency is affected by several factors such as electron hole effective mass, diffusion length, exciton lifetime, defects, band bending, surface band structure, thermal stability and photocorrosion^20^. To overcome these drawbacks, ZnO band structure was altered by several metals, non-metals and semiconductor coupling to extend its photo-response behavior into visible light region. Among them, semiconductor coupling is an efficient method to reduce the photo corrosion and rate of recombination of excitions. This leads to enhance the light harvesting ability up to the visible region without affecting its intrinsic character^6^. Recently several attempts were made in ZnO coupling with various narrow band gap semiconducting materials like ZnO-Fe_2_O_3_^21,22^, ZnO-WO_3_^21,22^, etc, to enhance the removal efficiency than bare ZnO under visible light irradiation.

Lead sulfide (PbS) has broad spectral response form visible to near-IR region due to its narrow direct band gap (0.41 eV). In recent years, PbS sensitized nanomaterial’s are increased the visible light response and photo-conversion efficiencies. It has unique photo physical properties such as multi exciton generation (MEG), high absorption coefficients, size dependent optical properties and optoelectronic properties^23,24^.

Recently, several methods are adopted for the preparation of ZnO-PbS heterojunction such as chemical bath deposition^25^, ultrasound deposition method^26^, low temperature method^27^, successive Ionic Layer Adsorption and Desorption method^28,29^, Radio Frequency Sputtering^30^ and spin coating method^31^. In all the above methods, most of them have some difficulties and limitations to produce ZnO-PbS heterojunction such as long reaction time and cost of the equipment. Therefore, novel techniques required to overcome this problem to prepare the semiconductor coupled ZnO-PbS photocatalyst in large-scale production without any sacrification in efficiency.

Microwave irradiation technique is a best alternative heating source for the preparation of nano materials due to its rapid chemical reaction with short span of time when compared to conventional methods. Microwave irradiation involves through dipolar polarization and ionic conduction, which helps for the preparation of nanostructured materials^32^. Microwave method is fast, uniform heat distribution, energy efficient and simple than other conventional methods. The size and morphology of the material can be easily controlled by the microwave parameters such as frequency, time and operating power. Therefore, microwave method is leading technique for homogeneous nucleation and fast crystallization, which leads to the preparation of nano-structured inorganic semiconductor photocatalyst. In recent years, different kinds of photocatalyst were prepared and reported such as InVO_4_^33^_,_ BiVO_4_^34^, CuO-Al_2_O_3_^35^, by the microwave irradiation.

In this study ZnO-PbS heterojunction was prepared under microwave irradiation method and their photocatalytic activities were investigated. The ZnO-PbS heterojunction improve the visible light absorption due to enhanced excitons life time through the charge transfer between the semiconductors interface. The structure, morphology and optical properties are characterized by XRD, FESEM, HR-TEM, XPS, PL, UV-Visible and EIS studies. The photocatalytic activities and mechanism of the as-prepared catalyst are studied with aniline and formaldehyde under simulated visible light and sun light. The degradation efficiency mainly depends on the transport of photo generated electron-hole pairs of ZnO-PbS interface quality. Thus, ZnO-PbS catalyst was optimized with different processing parameters.

## Results and Discussion

### XRD analysis

**Figure 1:**
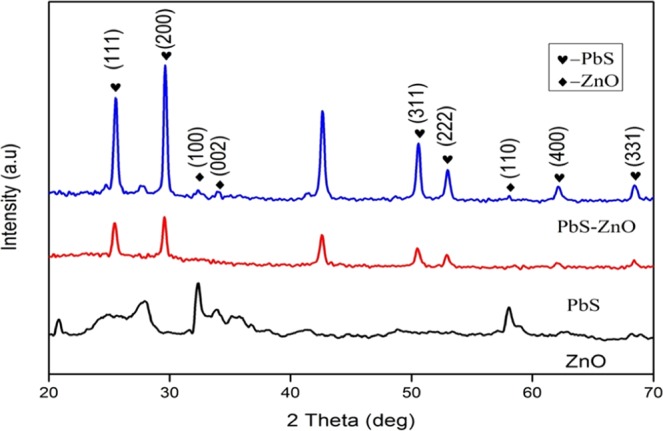
XRD patterns of ZnO, PbS and ZnO-PbS catalysts.

Figure1 represents the XRD patterns of ZnO, PbS and ZnO-PbS photocatalyst. The ZnO and PbS are exhibits hexagonal (wurtzite) and cubic structures matched in Joint Committee on Powder Diffraction Standards (JCPDS) card numbers 75–1526 (a = 3.22 and c = 5.28) and 03-0614 (a = 5.96) respectively. The characteristic peaks of ZnO (100), (002), (102) and (110) shows at the angles of 32.46°, 34.12°, 47.79° and 57.97° respectively. Also, the PbS diffraction peaks (111), (200), (311), (222), (400), (331) and (420) are observed at the angles of 25.55°, 29.61°, 50.57°, 52.98°, 62.13°, 68.40° and 70.51° respectively. The XRD spectrum of coupled ZnO-PbS exhibits both ZnO and PbS

peaks. Among them, PbS shows high intensity and more peaks than ZnO due to strong X-ray diffraction by PbS. It may be due to the PbS imprint over ZnO in accordance with FESEM and HR-TEM analysis. There is no other additional peaks were observed, which confirms the only presence of ZnO and PbS. The diffraction pattern also shows that the prepared photocatalyst are polycrystalline in nature. The average crystallite size of the ZnO, PbS and ZnO-PbS were calculated from XRD pattern using Debye-Scherrer formula Eq. (1)^36^

1 $${\rm{D}}=\alpha \lambda /\beta \,\cos \,\theta $$

where D is the average crystallite size, α is the scherrer constant (0.9), λ is the wavelength of the X-ray (0.154 nm), β is the full width at half maximum (FWHM) and θ is the Bragg angle of diffraction. Table (1) shows the average crystallite size calculated for the prominent peaks of ZnO, PbS and ZnO-PbS.

**Table 1:** Average crystallite size of ZnO, PbS and ZnO-PbS photocatalysts.

S.No	ZnO			PbS			ZnO-PbS		
1	2θ	β	D (nm)	2θ	β	D (nm)	2θ	β	D (nm)
2	32.46	0.003433	42.04	25.55	0.003433	41.39	25.5	0.00515	27.59
3	33.89	0.010299	14.06	29.61	0.003433	41.75	29.61	0.004291	33.40
4	47.79	0.017165	8.83	50.57	0.004291	35.71	32.46	0.010299	14.01
5	57.79	0.003433	46.10	52.98	0.00515	30.06	33.89	0.010299	14.06
6				62.13	0.010299	15.70	50.57	0.00515	29.76
7				68.4	0.006866	24.40	52.98	0.00515	30.06
8				70.51	0.00515	32.95	57.97	0.027465	5.76
9							62.13	0.006866	23.56
10							68.4	0.006008	27.88
11							70.51	0.00515	32.95
**Average**			**28**			**32**			**24**

### FE-SEM analysis

**Figure 2:**
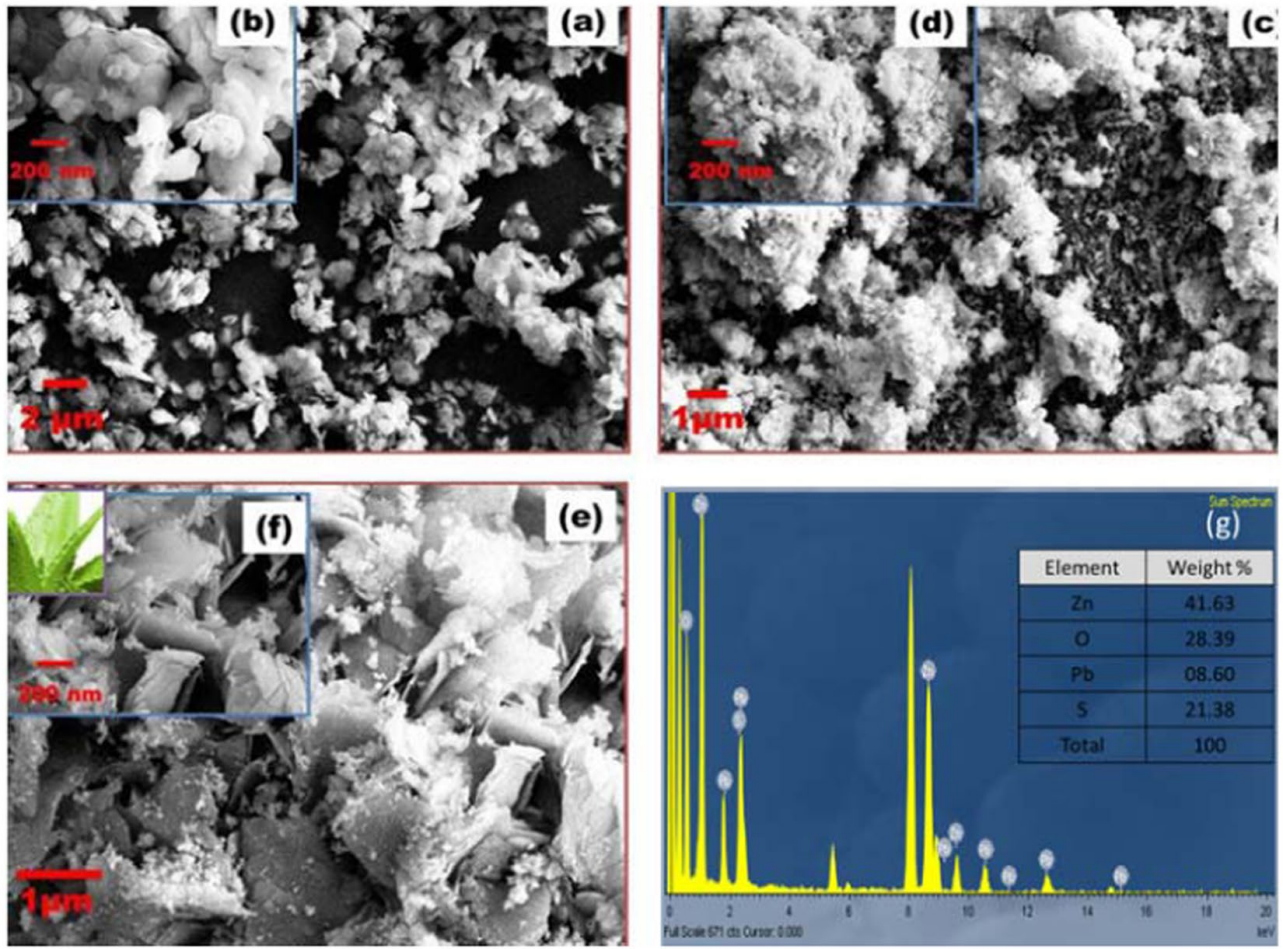
FESEM analysis of ZnO (**a**,**b**), PbS (**c**,**d**), ZnO-PbS (**e**,**f**) and EDS (**g**) analysis.

Figure 2 reveals the morphology of ZnO, PbS and ZnO-PbS photocatalyst using FESEM at different magnifications. The morphological characteristics of ZnO reveals nano-plate like structures oriented with different angles and size. The measured nano-plate diameter and thickness were ~75 nm and ~15 nm respectively (Fig. 2(a,b)). PbS shows sponge like morphology formed by the agglomeration of small particles with the size of ~50 nm (Fig. 2(c,d)).

Figure 2(e,f) shows the ZnO-PbS heterojunction nanostructure morphology. It exhibits the formation of smooth interlinked sheets with apparent spaces between the sheets and small particles are imprinted on nanosheet. In addition, bare ZnO nanoplates are changed into nanosheet like structure during ZnO-PbS formation, it may be due to the introduction of PbS. However, PbS size could not be varied for the formation of ZnO-PbS heterojunction. The average diameter of the sheet is approximately 500 nm with ~15 nm thickness and imprinted nanoparticle size ~50 nm. It was also identified that the sponge like structure of PbS imprinted over ZnO nanosheets as like water droplet over aloe-vera leaf.

The attained morphology of ZnO-PbS photocatalyst is suitable for producing reactive oxygen species. As photogenerated electron hole pair’s transaction could be very effective between the coupled ZnO-PbS photocatalyst under visible light^26^. Figure 2(g) shows the EDS spectra of the as-prepared photocatalyst. The EDS analysis indicates the presence of Zinc (Zn), oxygen (O), lead (Pb) and sulfide (S) elements in the ZnO-PbS photocatalyst. This confirms the formation of ZnO-PbS heterojunction in accordance with XRD.

### HRTEM analysis

**Figure 3:**
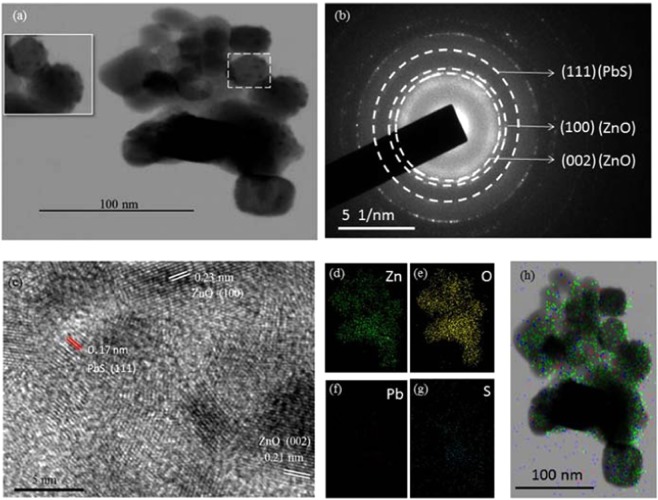
HRTEM analysis of ZnO-PbS heterojunction Image (**a**), SAED pattern (**b**), Lattice fringes (**c**), elemental mapping Zn (**d**), O (**e**), Pb (**f**), S (**g**) and ZnO-PbS (**h**).

The HRTEM reveals additional information about the crystalline, structural and elemental compositions details of the ZnO-PbS heterojunctions in Fig. 3. ZnO-PbS heterojunction exhibited in spherical like morphology with size of ~25 nm and small particles were decorated over the spherical structure which confirms the PbS decorated over the ZnO (insert) Fig. 3(a). Further, the selected area electron diffraction (SAED) pattern reveals the ZnO exist in (100) and (002) planes, also PbS exhibited in (111) plane which is confirms the ZnO-PbS heterojunction exist in polycrystalline nature which is well matched with XRD analysis in Fig. 3(b).

The ZnO-PbS heterojunction exhibited in 0.17, 0.21 and 0.23 nm lattice spacing of (111), (002) and (100) planes respectively Fig. 3(c). The elemental mapping confirms the presence of the Zn, O, Pb and S elements and distributed uniformly in the ZnO-PbS photocatalyst Fig. 3(d–h).

### XPS analysis

**Figure 4:**
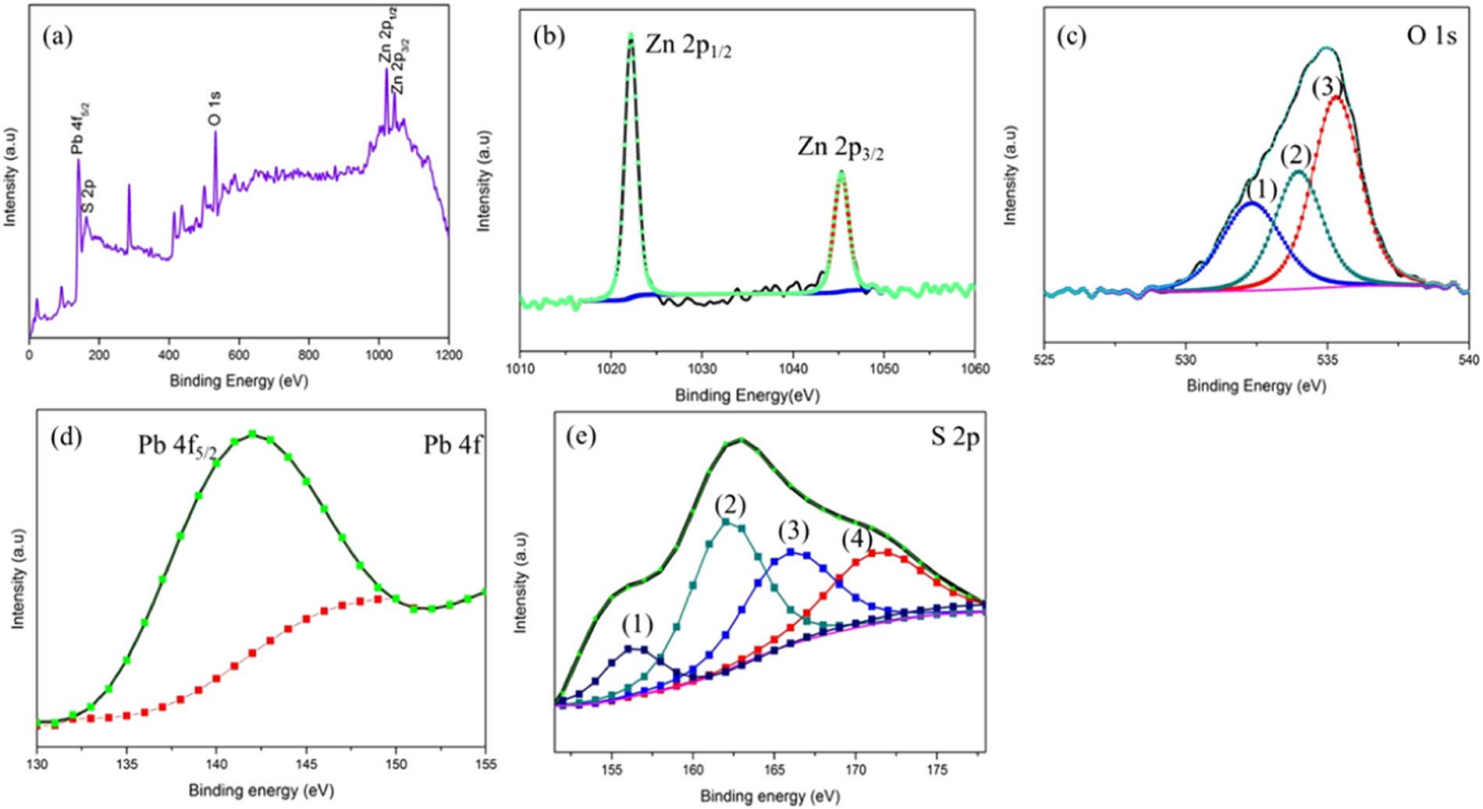
XPS analysis of ZnO-PbS heterojunction survey spectrum (**a**), core spectrum of Zn 2p (**b**), O1s (**c**), Pb 4 f (**d**) and S 2p (**e**).

The chemical composition formation of the ZnO-PbS photocatalyst was identified by XPS analysis (Fig. 4). The survey spectra of the semiconductor coupled ZnO-PbS photocatalyst exhibited the presence of Zn 2p, Pb 4 f, S 2p and O 1 s peaks in Fig. 4(a). Figure 4(b) shows two strong peaks at the binding energies of 1021.41 eV and 1045.34 eV for Zn 2p_1/2_ and Zn 2p_3/2_ respectively. The spin-orbital splitting value between the two strong peaks is 23.93 eV which confirms the Zn presence in the state of Zn^2+^^37^. The Fig. 4(c) shows the high resolution spectra of O 1 s Gaussian peak (1) at 532.80 eV confirms the presence of O^2^- ions in the lattice (O_L_). The peak (2) at 533 eV attributed for the Weakly bound surface oxygen. The peak (3) 535 eV assigned to O 2p state. These oxygen ions are chemisorbed with Zn^2+^ ions and formed ZnO in the hexagonal wurtzite structure in accordance with XRD results^38–40^. The high intensity characteristic peak of Pb 4f_5/2_ observed at 141.9 eV in Fig. 4(d). The characteristic peak (2) observed around 161.9 eV is attributed due to sulfide S 2p state binding energy and it indicates the formation of the PbS in cubic structure in accordance with XRD analysis^37^. The peak (3) at 165.8 eV assigned to S 2p_3/2_ which was confirmed the presence of ZnS due to the sulfur incorporate with ZnO^41^. The peak (4) at 171 eV may be assigned to strongly sulfite oxidized species (S^4+^)^42^ in Fig. 4(e). Thus, the XPS results indicate the successful formation of ZnO-PbS heterojunction.

### Optical properties

#### Photoluminescence property

**Figure 5:**
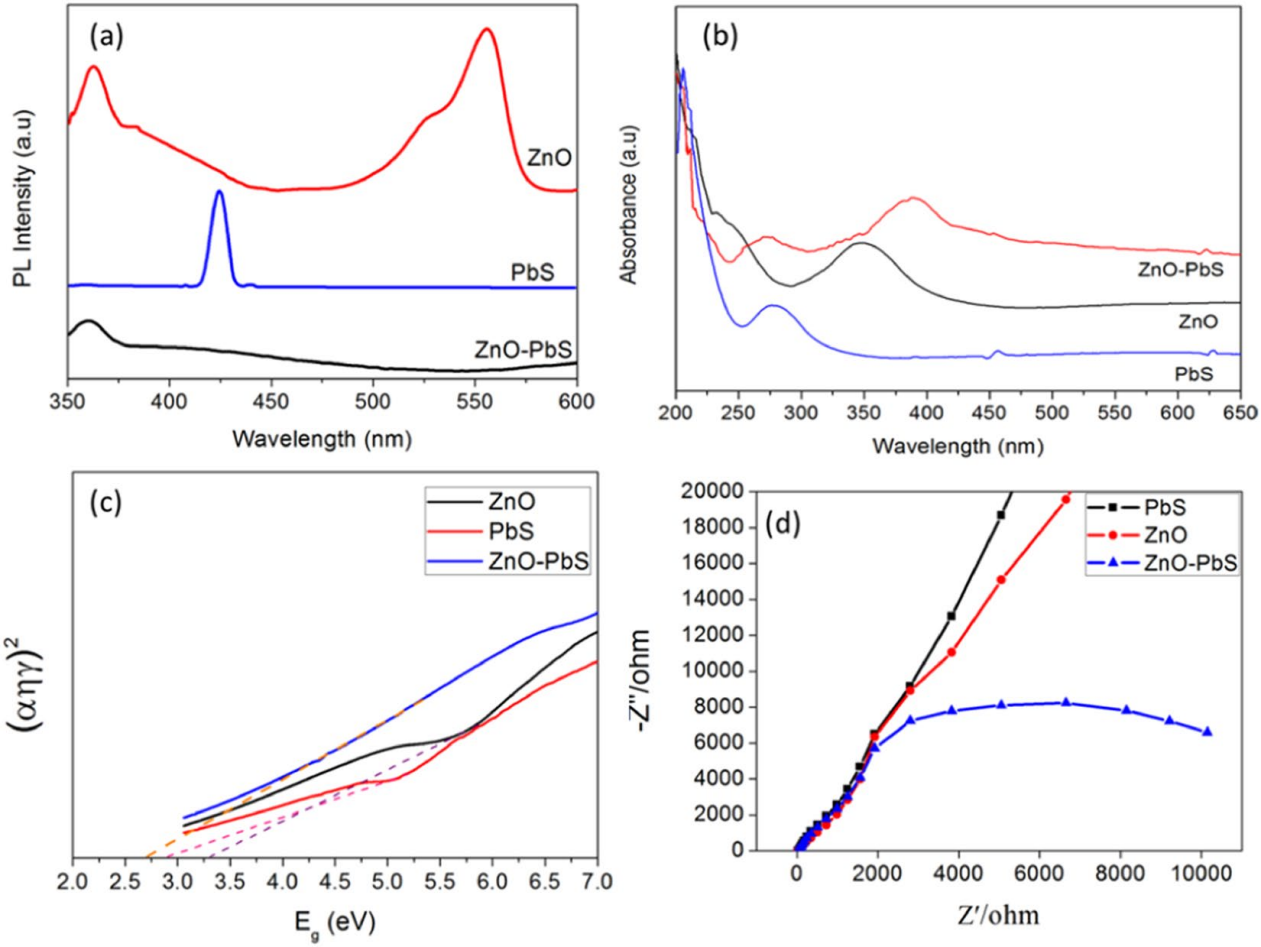
PL spectrum (**a**), UV-Visible absorbance spectrum (**b**), Tauc’s plot (**c**) and EIS (**d**) of ZnO, PbS and ZnO-PbS.

Electron-hole recombination process of ZnO-PbS photocatalyst was investigated through photoluminescence spectroscopy (PL). The PL emission intensity is mainly due to surface structural defect and high electron-hole recombination rate which can reduce the photocatalytic activity^43^. The PL spectra of ZnO, PbS and ZnO-PbS at room temperature were recorded for the excitation wavelength of 325 nm (Fig. 5(a)). The bare ZnO exhibited a sharp emission peaks at 360 nm and 387 nm whereas broad emission at 550 nm. The peaks at 360 nm, 387 nm and 550 nm indicates the near band edge emission, electron-hole pair’s recombination and defects (vacancies, interstitial and intrinsic) respectively^29,44^. As-prepared PbS exhibited narrow emission peak at 430 nm due the photo-generated electrons trapping by an interstitial Pb^2+^ sites. The trapping take place when the transition between conduction band edge to valance band edge of PbS^45^. The significant PL emissions were suppressed in the ZnO-PbS heterojunction than bare ZnO and PbS. The ZnO-PbS heterojunction shows the emission peak at 360 nm with decreased intensity than bare ZnO. It may be due to introduction of PbS nanoparticles over ZnO. The emission peak at 387 nm of ZnO is almost disappeared due to reduced rate of electron-hole recombination. This may leads to enhance the charge carrier transfer between ZnO and PbS. The emission peaks at 430 nm and 550 nm were also disappeared for the formation of ZnO-PbS heterojunction. Sulfur atoms are mainly occupied in the oxygen vacancies of ZnO surface due to sulfur and oxygen have similar chemical properties, thus the green emission peak (550 nm) was disappeared. Similarly, Pb atoms also have the tendency to occupy in the Zn vacancies. The occupancy of sulfur and Pb ions would initiate the nucleation of PbS over ZnO^29,46^. In addition, the formation of PbS may also alter the surface defects or create the new defects over ZnO. Therefore, the emission peaks at UV (387 nm) and green peak intensities (550 nm) were decreased or disappeared, which suggests that the electron-hole pairs were successfully transfer in- between ZnO and PbS. These charge transfer mechanisms suppresses the recombination rate of the excitons and enhance the degradation efficiency due to the formation of more radicals.

#### UV-visible analysis

Figure 5(b) shows the UV-Vis absorption spectra of ZnO, PbS and ZnO-PbS in the range of 200–650 nm range. The pure ZnO naoparticles shows broad absorption at 340 nm associated with the exciton transition. The PbS shows the absorbance peak at 284, 430 and 627 nm. The absorbance peak at 284 nm can be related to the crystal defects and 453 nm attributed to the transitions between the higher energy bands rather than exciton transitions. The absorbance peak at 627 nm may be related to exciton transitions and it has underwent massive blue-shift compared with bulk PbS absorption due to quantum confinement effects^45^. The ZnO-PbS absorbance spectrum shows absorbance peaks with reduced intensity at 287 nm and 453 nm. Also, ZnO absorbance peak was slightly shifted to higher wavelength (red-shift) due to interaction between ZnO and PbS ^29^. The presence of 627 nm absorbance peak indicates the ZnO-PbS heterojunction response towards visible light range. Therefore, the ZnO-PbS heterojunction reduces the crystal defect and enhances the visible light responses than bare PbS and ZnO respectively. The bandgap energy was calculated from the UV absorbance spectra. The bandgap value of ZnO and PbS was calculated from the tauc plot^47^

2 $$\alpha hv={\rm{A}}{(hv-{{\rm{E}}}_{{\rm{g}}})}^{{\rm{n}}}$$

where, α is absorption co-efficient, E_g_ is bandgap, hv is the photon energy, A is constant and n = 1/2 due to the direct optical transition in ZnO-PbS. The measured bandgap values are 3.3, 2.9 and 2.7 eV for ZnO, PbS and ZnO-PbS respectively in Fig. 5(c). The formation of the ZnO-PbS heterojunction enhances the visible light absorbance than bare ZnO.

The charge separation and electron transfer resistance of the ZnO, PbS and ZnO-PbS heterojunction were measured by electrochemical impedance spectroscopy. EIS Nyquist plots of the different photocatalyst shown in Fig. 5(d), the low radius arc indicates low electron transfer resistance. The ZnO-PbS heterojunction showed small curve and bent compared other catalysts such as ZnO and PbS. This resulted higher charge separation and lower electron transfer resistance for ZnO-PbS heterojunction^37^. The photo-generated electron and hole pairs were effectively separated by the introduction of PbS nanosponge over ZnO nanosheet to form the heterojunction which gives better photocatalytic degradation for organic pollutant.

### Photocatalytic degradation studies

#### Effect of the initial concentration

**Figure 6:**
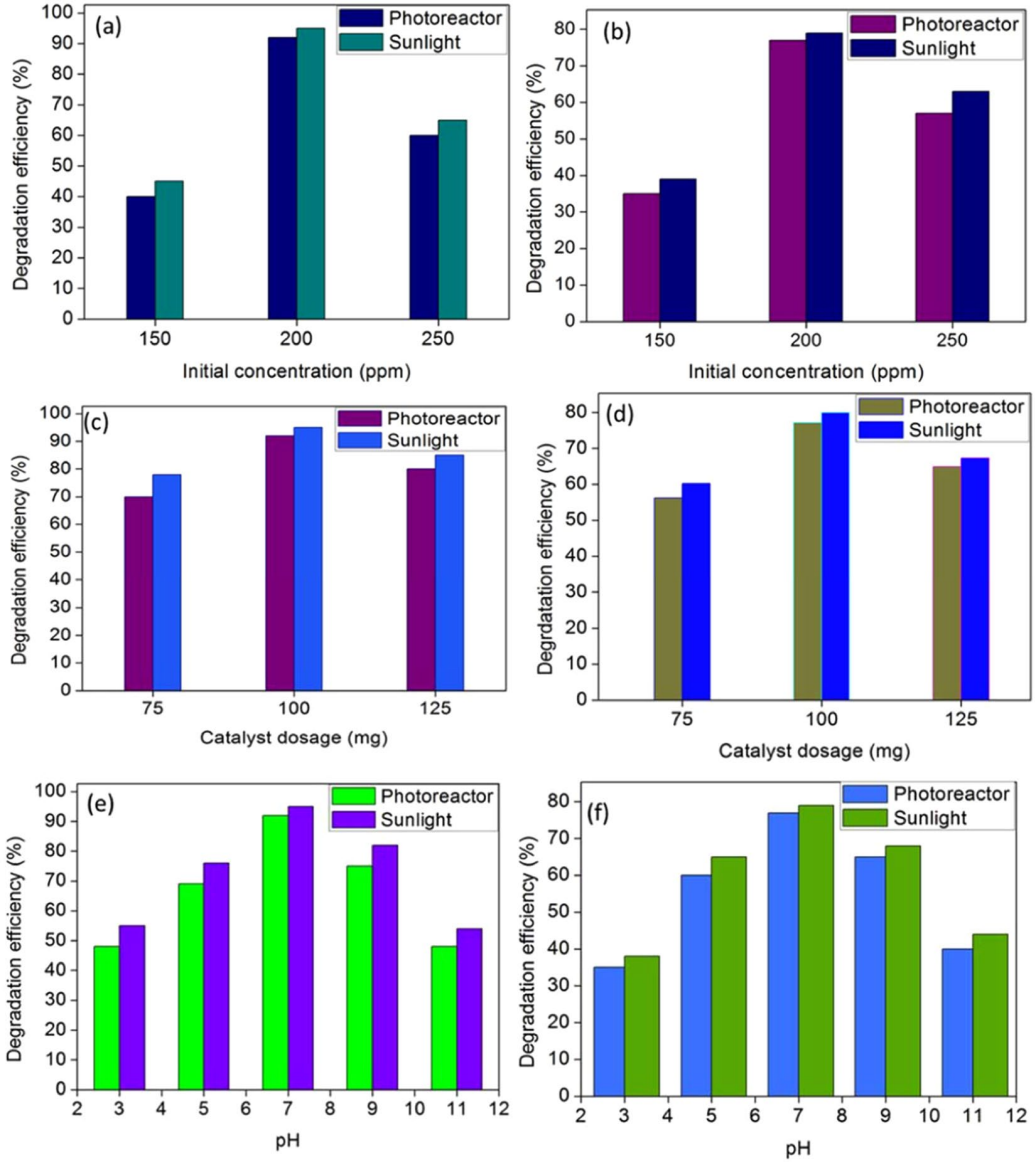
Optimized parameters of ZnO-PbS photocatalytic activity for aniline (**a**,**c**,**e**) and formaldehyde (**b**,**d**,**f**); Effect of initial concentration (**a**,**b**); Effect of catalyst dosage (**c**,**d**); Effect of pH (**e**,**f**).

100 ml of aniline and formaldehyde solution were prepared with different concentration (150, 200 and 250 ppm). The prepared solution maintained with pH 7 and it was investigated with 100 mg of ZnO-PbS catalyst. Fig. 6(a,b) shows the photocatalytic degradation of different organic pollutants varying with initial concentration. The degree of degradation efficiency of the organic pollutants gradually increases from 150 to 200 ppm and decreases from 200 to 250 ppm. Increasing degradation efficiency of the catalyst is due to more number of active sites existences than the organic molecules at lower concentration. At higher concentration of pollutant, the numbers of molecules were increased than the catalyst active sites which reduce the degradation efficiency of the reaction. Increasing organic molecules concentration restricts the light to reach the catalyst surface by shadow effect. Aniline exhibits higher degradation efficiency than formaldehyde.

#### The effect of photocatalyst dosage

The influence of catalyst dosage was also taking into observations by varying catalyst amount from 75 to 125 mg (Fig. 6(c,d)). The organic pollutants degradation was carried out with the concentration of 200 ppm at pH 7 by varying catalyst dosage. It was found that the degradation percentage was increased from 75 to 100 mg due to increasing photo-generated electron-hole pairs through effective light absorption. Above 100 to 150 mg of catalyst dosage effects decreased in the degradation percentage due to agglomeration of the particles, high turbidity and low scattering effect^48,49^.

#### The effect of pH of the solution

Figure 6(e,f) shows the effect of pH variation in the 100 mg catalyst dosage in 200 ppm under photocatalytic degradation of organic pollutants. The photocatalytic degradation efficiency of both the organic pollutants was increased when the pH varies from 3 to 7, whereas beyond pH 7 the degradation efficiency was decreased. Increasing the degradation efficiency may be due to electrostatic attraction between the catalyst and pollutants. In addition, the degradation efficiency was decreased above the neutral pH due to the electrostatic repulsion between the radicals and catalyst surface.

### Photocatalytic degradation of organic pollutants

#### Photocatalytic degradation of the pollutant under visible irradiation

**Figure 7:**
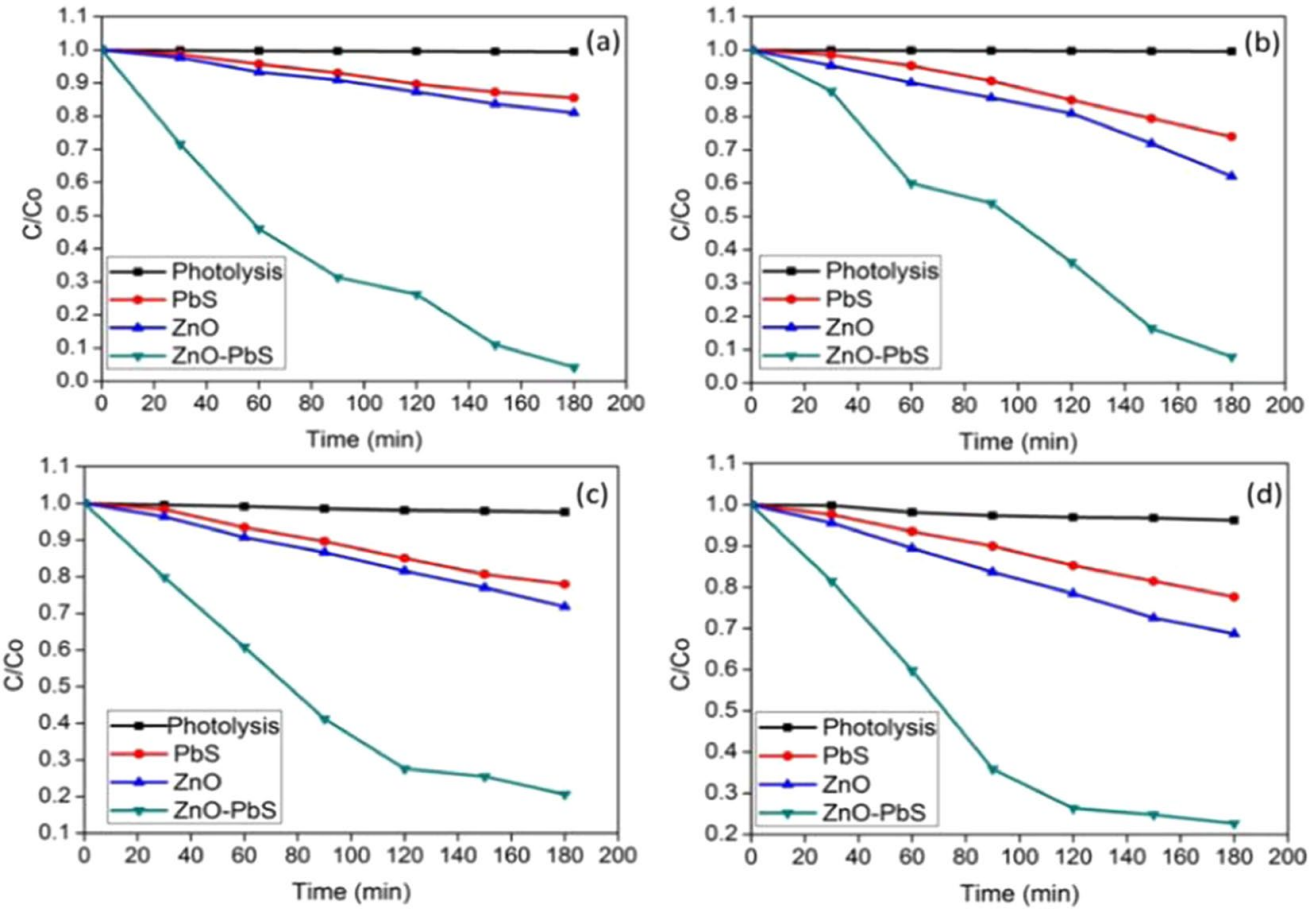
Photocatalytic degradation analysis of aniline (**a**,**b**) and formaldehyde (**c**,**d**) under sunlight (**a,c**) and visible light (**b,d**).

Figure 7( b,d) shows the photocatalytic activity of as prepared catalyst evaluation in different organic pollutants such as aniline and formaldehyde under visible light illumination. Absence of Self-degradation of aniline and formaldehyde was confirmed under visible light. Among the photocatalyst PbS exhibited low photocatalytic degradation efficiency than ZnO. The ZnO-PbS photocatalyst was successfully degraded around 92% and 77% of aniline and formaldehyde respectively within 180 min. The ZnO-PbS heterojunction shows higher photocatalytic degradation efficiency for aniline rather than formaldehyde.

#### Photocatalytic degradation of the pollutant under solar light irradiation

Figure 7(a,c) shows the photocatalytic activity of the ZnO-PbS catalyst against the organic pollutants of aniline and formaldehyde under sun light irradiation. The aniline and formaldehyde has higher degradation under solar light irradiation than visible light irradiation. The ZnO and PbS effectively harvest energy from solar spectrum as it consists both UV and visible light. The heterostructure catalysts also suppress the recombination rate of excitons, which helps to increase the amount of reactive oxygen species. The ZnO-PbS degrade the aniline and formaldehyde around 95% and 79% respectively under solar light. Table (2) shows the photocatalytic degradation efficiency of aniline and formaldehyde. Tables (3) and (4) show the comparison results with previous reported photocatalytic degradation efficiency for aniline and formaldehyde respectively. It is stated that the present research work exhibited higher degradation efficiency under higher concentration (200 ppm) with less time duration (3 hours).

**Table 2:** Photocatalytic degradation efficiency.

Catalysts	Photocatalytic degradation efficiency of Aniline (%) (200 ppm, 100 mg and pH-7)		Photocatalytic degradation efficiency of Formaldehyde (%) (200 ppm, 100 mg and pH-7)	
	Visible light	Sun Light	Visible light	Sun light
ZnO	21	35	25	29
PbS	10	27	17	20
Zno-PbS	92	95	77	79

**Table 3:** Comparison of photocatalytic aniline degradation.

Catalysts	Concentration mg/L	Light source	Reaction time duration (hours)	Degradation Efficiency (%)	References
Halloysite	10–40	Xenon 300 W	4	50.3–64	^50^
BiO_1.1_Br_0.8_	50	LED 18 watt	4	90	^51^
H_2_O_2_/TiO_2_	10	UV Hg lamp and sun light	2	85	^52^
Cr: ZnO	150–250	Sun light	6	74.5–93.3	^53^
TiO_2_/ZnO/HPMo	50	LED	3	25.6–43.4	^54^
Ag-AgBr/HHST	200	Tungsten (visible) and xenon (UV)	2.5	20–90	^12^
ZnO-PbS	150–250	Tungsten halogen 3oo Watt and sunlight	3	92–95	Present work

**Table 4:** Comparison of photocatalytic formaldehyde degradation.

Catalysts	Concentration mg/L	Light source and irradiation time	Reaction time duration (hours)	Degradation Efficiency (%)	References
Continues TiO_2_	10	UV lamp 25 W	14	96.4	^55^
V_2_O_5_/TiO_2_	180	Sunlight	4	89	^56^
Ag_3_PO_4_/TiO_2_	3.2	Xenon 350Watt	1.10	80	^57^
Fe doped WO_3_	2.4	LED 3.6 mW	6	98.2	^58^
TiO_2_/ACF-8h	0.8	UV lamp 8 Watt	2	83.6	^59^
TiMS2–550	6.56	UV lamp 20 watt	12	90	^60^
BiOBr/30% BiPO_4_	150	LED 7 watt	2	99	^61^
Au/TiO_2_	50±2	LED 19 mWatt		83.3	^62^
ZnO-PbS	200	Tungsten halogen 3oo Watt and sunlight	3	77–79	Present work

#### Reaction kinetics of photocatalytic degradation

**Figure 8:**
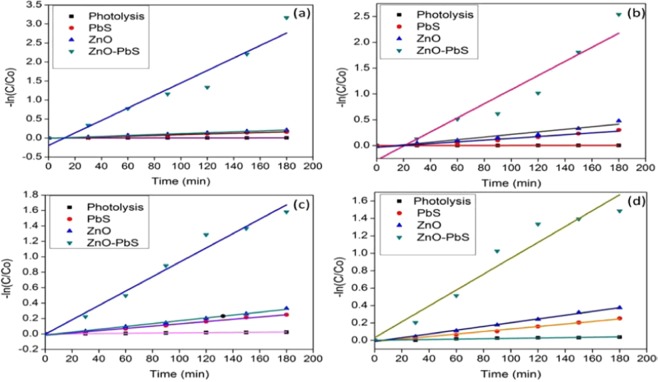
Reaction kinetics analysis of aniline (**a**,**b**) and formaldehyde (**c**,**d**) under sun light (**a**,**c**) and visible light (**b**,**d**) illumination with different photocatalyst.

The reaction kinetics of the prepared catalysts were investigated against organic pollutants under visible light by using modified Langmuir-Hinshelwood Equ. (3)

3 $${\rm{Kt}}=-\,\mathrm{ln}({\rm{C}}/{{\rm{C}}}_{{\rm{o}}})$$

where K is apparent rate constant, t is reaction time, C and C_o_ is the final and initial concentration of the organic pollutants. Figure 8 shows the reaction kinetics profile for the ZnO, PbS and nanostructure ZnO-PbS catalysts. The straight line indicates photocatalytic degradation which follows pseudo first order reaction kinetics with respect to pollutant concentrations. The Table (5) shows all catalysts correlation coefficient and apparent rate constant in the degradation of the organic pollutants. Among the catalysts, ZnO-PbS heterojunction exhibits highest rate constant for the degradation of organic pollutants.

**Table 5:** Comparison of reaction kinetics parameters

S.No	Catalyst	Aniline				Formaldehyde			
		Visible light		Sun light		Visible light		Sun light	
		R^2^	K	R^2^	K	R^2^	K	R^2^	K
1	Photolysis	0.9319	0.0002	0.9262	0.0001	0.9902	0.0002	0.9792	0.0001
2	PbS	0.9025	0.0017	0.9897	0.0009	0.9963	0.0014	0.9872	0.0014
3	ZnO	0.9935	0.0025	0.9954	0.0012	0.9330	0.0021	0.9933	0.0018
4	ZnO-PbS	0.9560	0.0136	0.9313	0.0164	0.9124	0.0091	0.9744	0.0093

#### Reusability of the photocatalyst

**Figure 9:**
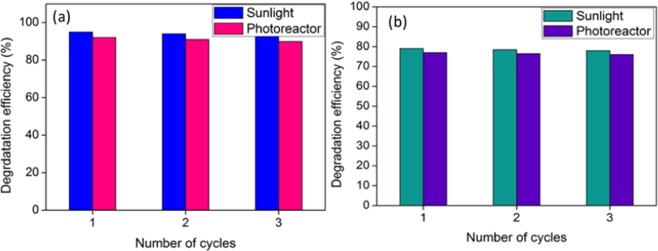
Reusability analysis of ZnO-PbS catalyst under sun light and visible light illumination of aniline (**a**) and formaldehyde (**b**).

The reusability of ZnO-PbS photocatalyst was investigated for the degradation of pollutant under visible and solar light irradiation. The photocatalyst were separated by centrifuge and washed with double distilled water for several times. The separated catalysts were dried and used for next run. The Fig. 9(a,b) shows the aniline and formaldehyde degradation successively tested for three cycles under visible and sunlight. The results show the efficiency of the photacatalytic reaction was decreased when the number of cycles increased. The small variation in the degradation percentage of organic pollutant is may be due to the loss of photocatalyst during separation process. The ZnO-PbS photocatalyst was stable up to 3 cycles.

#### HPLC analysis of photocatalytic degraded organic pollutants

**Figure 10:**
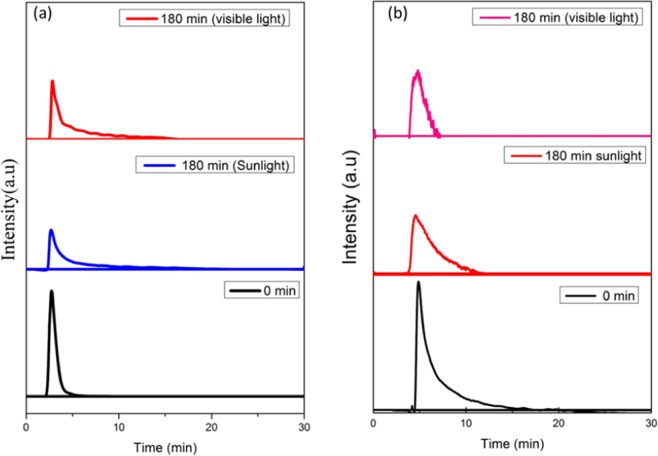
HPLC analysis of aniline (**a**) and formaldehyde (**b**) before and after visible and sun light illumination.

HPLC analyses were used to confirm the degradation of the organic pollutant under visible and solar light irradiations. The organic pollutants were collected before and after treatment (Fig. 10(a,b)). The chromatogram results confirms the degradation of the organic pollutants. Since, aniline and formaldehyde shows high degradation under sunlight than visible light irradiation at 180 min. However, aniline shows maximum degradation than formaldehyde under sunlight. The retention time of aniline and formaldehyde were 2.8 and 4.8 min respectively. The intensities of the chromatographic peaks were drastically decreased in both pollutants. The photocatalytic degradation of aniline and formaldehyde were turns in to some organic derivatives such as water, CO_2_ and mineral salts.

### The reaction mechanism

**Figure 11:**
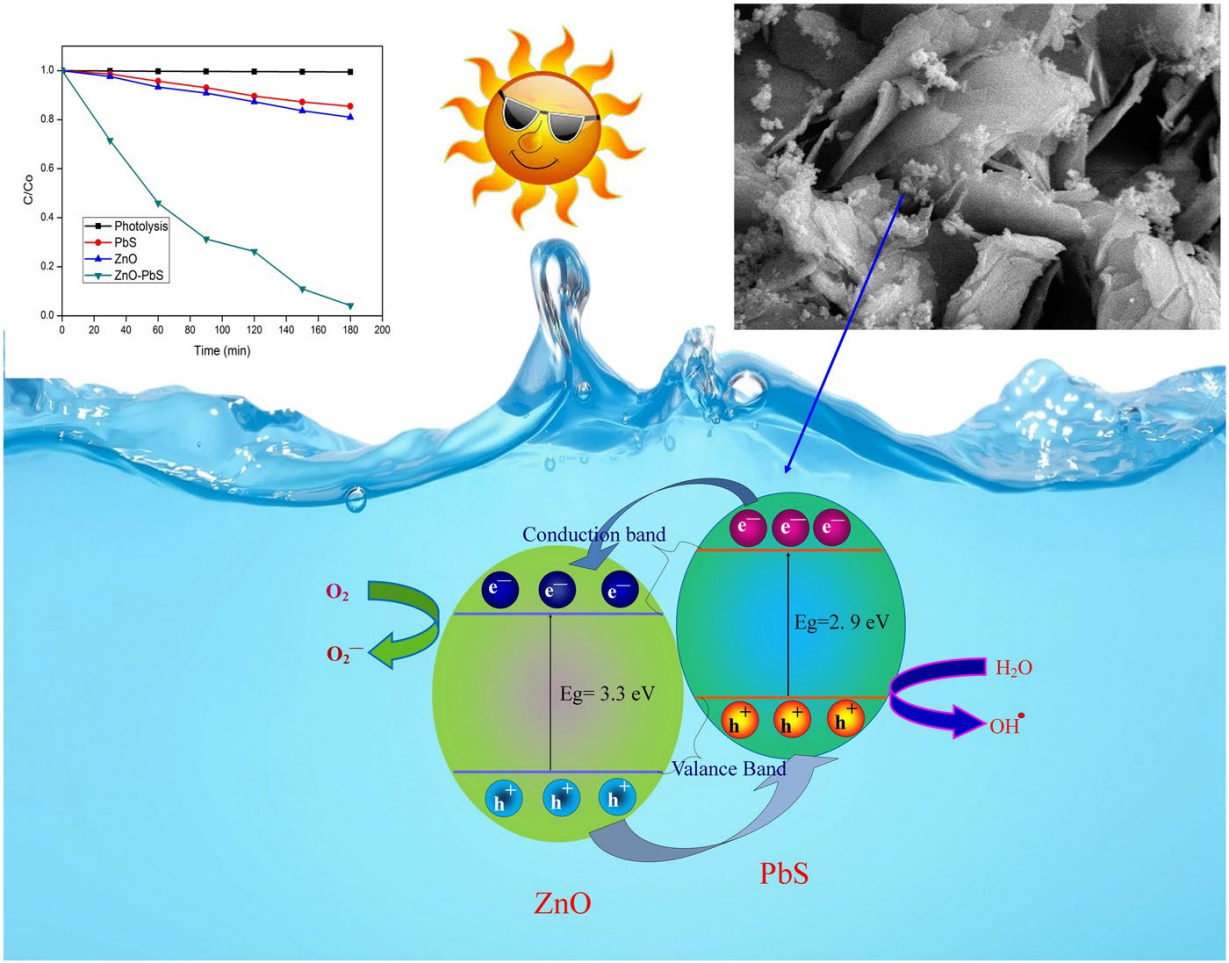
Possible photocatalytic reaction mechanism of ZnO-PbS heterojunction.

The photocatalytic activity of the ZnO, PbS and ZnO-PbS catalysts were examined. It was found that the synergistic effect of ZnO-PbS heterostructure leads to enhance the efficiency in the removal of organic pollutants than bare ZnO and PbS. Figure 11 shows the reaction mechanism of the organic pollutants degradation. The bare ZnO bandgap energy (3.3 eV) is majorly response for the ultraviolet region. However, the bare PbS measured bandgap energy (2.9 eV) supports for the visible region in the solar spectrum. The PbS conduction band placed over ZnO conduction band and the valance band of PbS placed in between of ZnO Cb and Vb when ZnO-PbS heterojuction formation. In the case of visible irradiation, ZnO acts as an electron extractor from the PbS conduction band due to ZnO bandgap limitations. Hence, the life time of the charge carriers are increased due to wide bandgap of ZnO.

In the case of solar irradiation, sunlight consist of 4% UV and 47% visible region. The photo generated electrons in the conduction band of PbS transfer into ZnO conduction band. In addition, ZnO also produce the electron-hole pairs under UV illumination. Photo generated holes were moves from ZnO to PbS valance band. Therefore, photo generated electrons and holes mobility were increased by solar irradiation. In addition, the photo generated electron-hole pairs are effectively transports between PbS and ZnO which reduce the recombination of charge carriers. The redox reactions produce different kinds of reactive oxygen species such as super oxide anion radical (O_2_^−^) and hydroxyl radical (OH·). These reactive oxygen species react with organic pollutant and convert or eliminate toxic into non-toxic compounds.

### Conclusion

In this investigation, we successfully prepared ZnO-PbS heterojunction through microwave irradiation technique for removal of aniline and formaldehyde under visible light irradiation. ZnO-PbS heterojunction were enhanced the visible light response and reduce the rate of charge carriers recombination process. Low electron transfer resistance of ZnO-PbS heterojunction improves the charge carrier transport between the semiconductors interface. The photocatalytic degradations were recorded around 95% and 79% for the aniline and formaldehyde respectively under solar spectrum when compared with previous report. Higher catalytic activities of ZnO-PbS heterojunction were stable upto 3 cycles (200 ppm, 100 mg and pH-7) The HPLC analysis were proved that the organic pollutants successfully mineralized into water, CO_2_ and mineral salts.

## Experimental Methods

### Synthesis of ZnO-PbS photocatalyst

The analytical grade chemicals such as zinc sulfate (ZnSO_4_.7H_2_O), potassium hydroxide (KOH), lead acetate (Pb(C_2_H_3_O_2_)_2_) sodium sulfide (Na_2_S), aniline (C_6_H_5_NH_2_), formaldehyde (CH_2_O) and ethanol (C_2_H_5_O_6_) were purchased from sigma aldrich chemicals. Double distilled water used as a solvent in all the experiments.

Initially, 25 ml of 0.1 M ZnSO_4_.7H_2_O and 25 ml of 0.1 M KOH solutions were prepared using double distilled water, then added together and stirred vigorously for 10 minutes at room temperature. Pearl white color solution was formed. Same experimental conditions were maintained for preparing 25 ml of 0.1 M Pb(C_2_H_3_O_2_)_2_ and 25 ml of 0.1 M Na_2_S solutions which forms greenish yellow color solution. These two solutions were mixed together in the reaction flask and continuously stirred for 30 min. at ambient condition (pressure and temperature) and solution becomes black color. The reaction flask was placed in the microwave oven to carry out further reaction under the influence of microwave irradiation for 2 min. Then the reaction flask was allowed to cool at room temperature. A black colored product was obtained after filtered using filter paper. The product washed several times by double distilled water and ethanol to remove the residues. The final product was dried in hot air oven at 60 °C for 2 hours. The same procedure was followed for the synthesis of bare ZnO and PbS.

### Characterization

The formation of crystalline phases and structure of the composites were studied using X-ray diffractometer (Xpert-Pro) using CuKα radiation (λ = 1.54Ȧ) with operating voltage of 40 kV. Zeiss Field Emission Scanning Electron Microscopy (FESEM) with an operating voltage at 10 kV was used to reveal the morphology of ZnO-PbS heterojunction. HR-TEM analysis was carried out using Shimadzu. X-ray Photoelectron Spectroscopy (XPS, Thermo K-alpha) was used to analyze the elemental composition. The absorbance spectra and emission properties of the as-prepared photocatalyst were analyzed at room temperature using UV-Visible spectrophotometer (Lambda 35) and photoluminescence spectroscopy (PL) with excitation wavelength 325 nm (Horiba LabRAM HR-PL) respectively. The electrochemical impedance spectra of the photocatalyst were analyzed by Princeton Applied Research. The organic pollutant absorbance spectra were measured using UV-Visible spectrophotometer (Jasco V-650).

### Photocatalytic degradation of aniline and formaldehyde

Aniline and formaldehyde organic compounds were used as pollutants to investigate the degradation efficiency of as-prepared photocatalyst. The degradation tests were carried out in 250 ml beaker filled with 100 ml of aniline and formaldehyde aqueous solution separately. 100 mg of as-prepared photocatalyst were dispersed in both the solutions of aniline and formaldehyde. The solutions were placed under dark condition for 30 min to attain the adsorption-desorption equilibrium between the photocatalyst and pollutants. Then the solutions were irradiated by a 300 W tungsten-halogen lamp (osram). Similarly, solutions were also irradiated on a clear solar atmospheric condition between 10.00 a.m. to 3.00 p.m (May). During the degradation process, 1.5 ml of samples was collected with regular time interval. The collected samples were centrifuged at 1000 rpm to separate the photocatalyst from the solution. The degradation of the organic pollutants was examined by UV-Visible spectrophotometer. The photo-degradation efficiency of the ZnO, PbS and ZnO-PbS photocatalysts were evaluated under organic pollutants through the following relation Equ. (4)^44^

4 $${\rm{\eta }}( \% )=({{\rm{C}}}_{{\rm{o}}}/{{\rm{C}}}_{{\rm{e}}})/{{\rm{C}}}_{{\rm{e}}})\times 100$$

where η (%) = photo-degradation efficiency, C_o_ and C_e_ (mg/L) are the initial and final concentration of the organic pollutants respectively.

The stability of the as-prepared ZnO-PbS photocatalyst was investigated by re-cycling the experiment. After the light irradiation at particular time, the ZnO-PbS photocatalyst were collected from the solution by centrifuge technique. Further, the photocatalysts were washed several times using deionized water and ethanol to remove the residues of the organic pollutants. The photocatalysts were dried at 373 K for 2 hours and recycled which helps to reduce the preparation time, chemicals and cost of the photocatalyst. The efficiency of the photocatalytic reaction were analyzed with different parameters such as by pollutant concentrations, pH and catalyst dosage.

### High performance liquid chromatography analysis (HPLC) of photocatalytic degradation of organic pollutants

Photocatalytic degradation byproducts of aniline and formaldehyde were analyzed by high performance liquid chromatography (Shimadzu-UV-Vis detector) equipped with C-18 column. The methanol and water (50:50) were used as mobile phase for analyze of aniline. A mixture of acetonitrile and water (30:70) was used as mobile phase to resolve formaldehyde derivatives. 1 µl solutions of aniline and formaldehyde (before and after treatment) were injected into C-18 column and analyzed by UV detector.

## Data Availability

Readers can access based on the request to the authors.
